# Influence of Composition on the Patterns of Electrokinetic Potential of Thermosensitive N-(Isopropyl)Acrylamide Derivatives with Poly(Ethylene Glycol) Dimethacrylate and N-(2-Hydroxyethyl)Acrylamide

**DOI:** 10.3390/ijms252413554

**Published:** 2024-12-18

**Authors:** Monika Gasztych, Aleksandra Malamis-Stanowska, Mateusz Trafalski, Witold Musiał

**Affiliations:** 1 Department of Physical Chemistry and Biophysics, Pharmaceutical Faculty, Wroclaw Medical University, Borowska 211, 50-556 Wroclaw, Poland; monika.gasztych@umw.edu.pl (M.G.); amalamiss@gmail.com (A.M.-S.); 2 Department of Dental Surgery, Wroclaw Medical University, Krakowska 26, 50-425 Wroclaw, Poland; mateusz.trafalski@umw.edu.pl

**Keywords:** NIPA—N-isopropyl acrylamide, thermosensitive nanoparticles, hydrodynamic diameter, electrokinetic potential

## Abstract

The synthesis of poly(N-isopropyl acrylamide) (pNIPA)-based polymers via the surfactant-free precipitation polymerization (SFPP) method produced thermosensitive nanospheres with a range of distinctive physicochemical properties. Nano- and microparticles were generated using various initiators, significantly influencing particle characteristics, including the hydrodynamic diameter (D_H_), which varied from 87.7 nm to 1618.1 nm. Initiators, such as potassium persulfate and 2,2′-azobis(2-methylpropionamidine) dihydrochloride, conferred anionic and cationic functionalities, respectively, impacting the electrokinetic potential (EP) of the particles. Notably, certain particles with cationic initiators exhibited negative EP values at 18 °C, attributed to residual initiator components that affected the surface charge distribution. The presence of hydrophilic N-(2-hydroxyethyl)acrylamide (HEAA) segments also influenced solubility and phase transition behaviors, with critical dependencies on the HEAA/NIPA (N-isopropyl acrylamide) molar ratios. EP measurements taken at 18 °C and 42 °C revealed substantial differences, primarily governed by the initiator type and polymer composition. Observed variations in particle stability and size were associated with the choice of crosslinking agents and comonomer content, which affected both D_H_ and EP in distinct ways. This study provides insights into key factors influencing colloidal stability and electrostatic interactions within thermosensitive polymer systems, underscoring their potential applications in biomedical and industrial fields.

## 1. Introduction

### 1.1. Nanoparticles in Pharmaceutical Applications

Pharmaceutical development is largely focused on the search for new molecules with potential therapeutic effects. However, significant research is also dedicated to improving drug delivery methods that have been known for years. Advances in dosage form technology primarily aim to make it easier for patients to take their medications [1]. Nanoparticles have become increasingly important in modern science due to their unique properties and wide range of applications across various fields [2]. Because nanoparticles can be engineered to target specific cells and tissues, they hold great potential for medical applications, such as drug delivery, imaging, and cancer therapy [3]. One approach to achieving modified drug release is through the use of polymers that are sensitive to external stimuli. Various signals can influence the release of substances from such structures including temperature, light, pH, mechanical pressure, the presence of specific chemical compounds, or an electrical pulse [4]. Thermosensitive polymers belong to the group “smart polymers”. These compounds are often characterized by biocompatibility, which makes them suitable for many biomedical applications, including tissue engineering, controlled drug delivery systems (DDS), and the thermal modulation of enzyme functions [5,6,7]. They are also widely used in dentistry to support restorative procedures, implantology, orthodontics, prosthetics, and conservative dentistry [8]. Thermosensitive polymers are used in the manufacture of membranes for guided bone regeneration, dental pulp regeneration, drug delivery systems in periodontology, as well as in the manufacture of dental prosthetics, fillings, and adhesives [9,10,11,12]. They are also used as antibacterial polymer coatings to prevent bacterial growth on the surfaces of dentures and fillings, thereby extending their life [13]. A clinically important group of stimuli-responsive polymers are thermosensitive structures, which can be triggered to release the drug at either a low or high temperature [14]. Thermosensitive polymers are a notable class within polymer science, exemplified by N-isopropyl acrylamide (NIPA) and its derivatives. NIPA has a low critical phase transformation temperature, typically around 33 °C, which classifies it as a negatively thermosensitive material that contracts with increasing ambient temperature. Beyond its biomedical applications, NIPA demonstrates biocompatibility by avoiding immunogenic responses in biological environments, though its non-biodegradable nature limits its utility in internal therapeutic contexts [15].

### 1.2. Thermosensitivity

**Figure 1:**
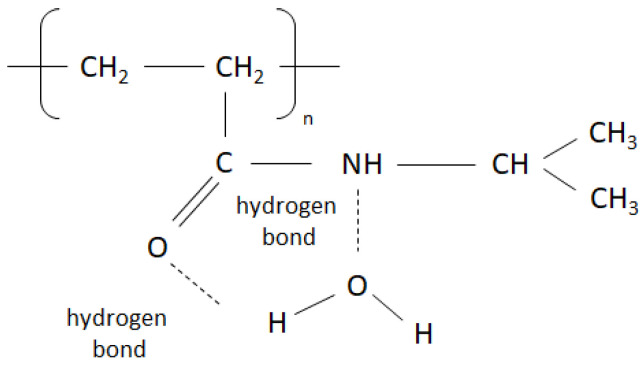
Chemical structure of a NIPA N-isopropyl acrylamide monomer. The central amide group enhances water solubility through hydrogen bonding, counteracting the hydrophobic characteristics of the chain backbone and side group [18].

The phase transition temperature of NIPA-derived polymers is modifiable by copolymerization with various comonomers and the incorporation of different substituents. For instance, the introduction of hydrophobic groups, such as butyl methacrylate, can lower the critical temperature, while the incorporation of hydrophilic groups, such as acrylamide, can increase it. Additionally, the presence of ionizable groups, such as carboxyl groups, makes the transition temperature pH-dependent, allowing volume changes to be made at a constant temperature with minor pH adjustments. These properties highlight the versatility of NIPA-based polymers in tailor-made applications that require precise environmental responses [16,17]. NIPA is a neutral polymer known for its unique amphiphilic properties, which arise from the presence of both hydrophilic and hydrophobic groups in its monomer structure. When the temperature is sufficiently low, water molecules form strong hydrogen bonds with the central amide groups (Figure 1).

### 1.3. Electrokinetic Potential

The electrokinetic potential, EP is an important parameter in the electrochemistry and the physicochemistry of colloids [19]. The surface charge characteristic of nanoparticles is a critical factor in targeted drug delivery. The zeta potential enables the evaluation of the stability of nanofluids and colloidal systems [20]. The modification of nanodrug delivery systems is the most common strategy to control the opsonization process and, thereby, to prolong the circulation time of the systems in the bloodstream [21,22]. EP has a major influence on the physicochemical properties of drug delivery systems, due to the formation of an electrical layers around each particle [23]. The potential at the boundary called the hydrodynamic shear surface or slip plane is identified as the EP [24]. The EP of stable dispersions is generally considered to exceed the module of 30 mV [25,26]. pH influences the surface charge of the particles through the protonation and deprotonation of functional groups [27]. A change in pH can result in an increase or decrease in surface charge, which in turn affects the value of the EP. Also, the temperature, which affects the mobility of the ions and their adsorption on the surface of the particles, may moderate the EP level at higher temperatures. The evaluation of these factors is crucial for the control of colloids stability, a vital factor in the material science of medical and pharmaceutical products [28].

### 1.4. The Identified Hydrodynamic Diameters

In our former studies, the number of thermosensitive derivatives has been partially characterized in terms of hydrodynamic diameter [29,30,31]. These dispersed data are presented together in Table 1.

### 1.5. The Aim

Polymer compositions from previous studies have already been published by our scientific group. In this paper, selected physicochemical studies of synthesized particles are expanded in the field of electrokinetic potential. We studied the influence of anionic and cationic initiators on the resulting EP and pH of thermosensitive NIPA derivatives with poly(ethylene glycol) dimethacrylate or N-(2-hydroxyethyl)acrylamide, or both, assessed at temperatures between 18 °C and 42 °C. Also, the influence of composition on the hydrodynamic diameter and polydispersity index (PDI) values of the synthesized microparticles was studied. Thus, the aim of this research was to investigate the effect of the composition of synthesized thermosensitive particles on the recorded EP in the context of pH, volume phase transition temperature (VPTT), and particle size.

## 2. Results

### 2.1. The Products of the Synthesis

The synthesis gave transparent solutions of polymers, which, after purification via equilibrium dialysis, presented some turbidity when heated. The turbidity disappeared after cooling. The freeze-dried product was a white powder.

### 2.2. Evaluation of pH and Electrokinetic Potential (EP)

A pH test was performed on all components used for particle synthesis and the results are presented in Table 2 Their pH was in the range 4.23–6.23.

The results of the pH test and the EP of the polymers at 18 °C and 42 °C are presented in Table 3. The pH measurements were carried out before (pH_BPP_) and after (pH_APP_) the purification process. Before purification, the pH was between 2.43 and 6.21, while, after purification, the pH values ranged from 3.42 to 9.05. The EP at 18 °C ranged from −14.87 mV to −4.97 mV with the use of an anionic initiator. On the other hand, the cationic initiator resulted in values of EP ranging from 9.07 mV to −3.06 mV. The EP values increased at an elevated temperature of 42 °C. The EPs of the anionic initiator group ranged from −28.97 mV to −21.1 mV. After using a cationic, alkaline initiator, the EPs ranged from 23.00 mV to 30.4 mV.

### 2.3. Evaluation of Hydrodynamic Diameter (D_H_), Polydispersity Index (PDI), and Volume Phase Transition Temperature (VPTT)

**Figure 2:**
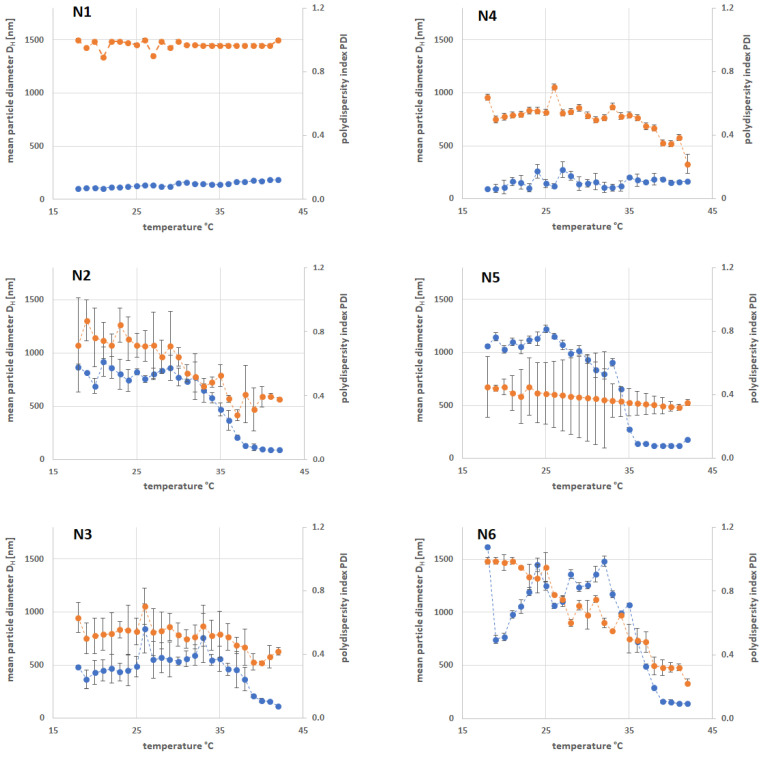
The effect of the temperature on the polydispersity index (PDI)—● and hydrodynamic diameter (D_H_)—● of the synthesized particles N1–N6 obtained from dynamic light scattering.

The results of D_H_ at 18 and 42 °C are presented in Table 1. In contrast, the exact D_H_ and PDI measurements as a function of temperature range are shown in Figure 2. Due to the data, the D_H_ at 18 °C of the particles obtained by surfactant-free precipitation polymerization SFPP with the anionic initiator ranged from 101.50 nm to 868.10 nm. However, when the cationic initiator was used, the D_H_ was characterized by varied values ranging from 92.39 nm to 1618.10 nm. The D_H_ versus the temperature curves of polymer samples N2, N3, N5, and N6 had similar change profiles with sharp rises, reflecting the phase transformation temperature (VPTT). In the case of N1 and N4 particles, we do not observe a clear VPTT. These are particles that show an increase in D_H_ under the influence of the temperature increase, which could be due to the aggregation process.

The DLS measurements were based on statistical methods, and to obtain more comparable information, the PDI was determined by DLS. At 18 °C, a low PDI value of 0.45 was found for the N5 particles; whereas, the highest PDI value of 1.0 was observed for N4. At 42 °C, the PDI of N1 exhibited a value of 1.00. At temperatures above the VPTT of 42 °C, a D_H_ range of 87.70 nm–185.33 nm was observed. However, the value of D_H_ was significantly lower after the system was heated. High PDI values of 1.00 were observed for the N1 particles and can be associated with particle aggregation. These data are presented in Table 1, in accordance with the VPTT in Table 4 excavated from the continuous measurement of D_H_ in the course of the increasing temperature.

The observed values of VPTT were in the range 32–38, as it is presented in Table 4.

## 3. Discussion

### 3.1. General Remarks on the Properties of the Synthesized Macromoecules

**Figure 3:**
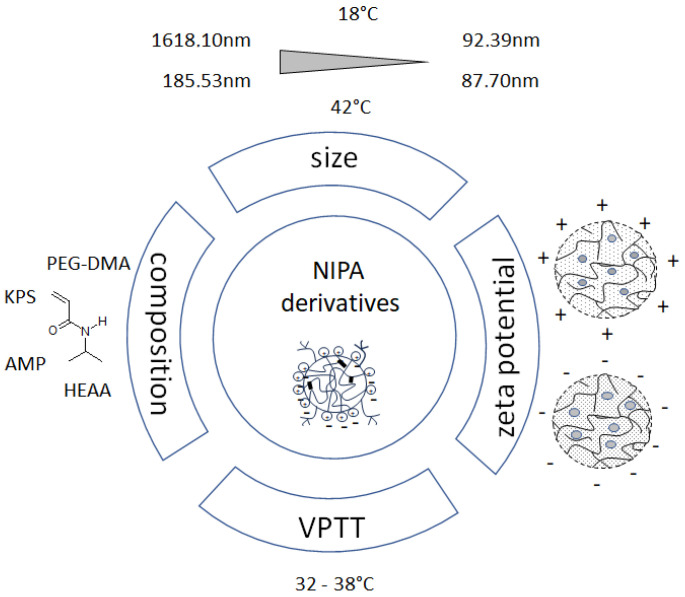
Schematic diagram of the methods and abbreviated results used in the present work—measurement of particle size, zeta potential, and phase transition temperature.

SFPP is a commonly used method for the synthesis of pNIPA-based polymers. It successfully allowed us to obtain thermosensitive nanospheres with different properties [32]. In a series of syntheses, different types of minor nano- and microparticles were produced. A schematic diagram of the applied methods and the abbreviated results used in the manuscript are shown in Figure 3.

### 3.2. Variability of pH and Electrokinetic Potential

#### 3.2.1. Environmental pH

**Figure 4:**
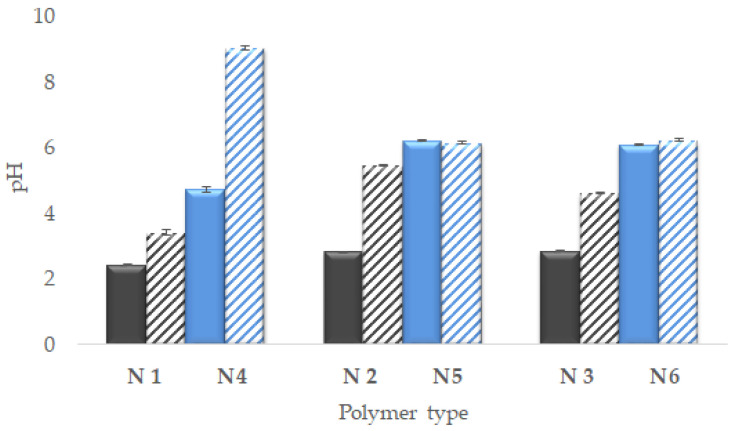
The variability of the pH values of particles N1–N6, polymers synthesized with the anionic initiator (black columns, left side), and polymers synthesized with the cationic initiator (blue col-umns). Full columns are the pH values before purification, and gradient columns are the pH values after purification.

The variability of the pH of particles N1 to N6 before and after purification is shown in Figure 4.

**Figure 5:**
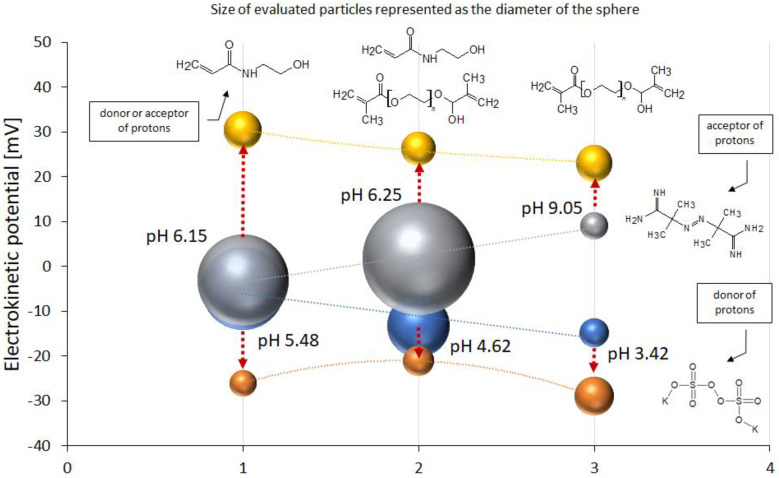
The electrokinetic potential, pH, and size of assessed polymeric particles. The red dotted arrows represent the increase in temperature from 18 to 42 °C. Type of particle: at 18 °C: (1)-N2 (blue) and N5 (grey); (2)-N3 (blue) and N6 (grey); (3)-N1 (blue) and N4 (grey); at 42 °C: (1)-N2 (orange) and N5 (yellow); (2)-N3 (orange) and N6 (yellow); (3)-N1 (orange) and N4 (yellow).

The pH changes were useful for assessing the degree of purification of the compounds. However, more interesting information may be excavated from the pH of the polymer solution after purification. We hypothesized that both the functional groups from the initiator and from the comonomer have an influence on the level of the hydrogen ions, protons in the aqueous dispersion of synthesized, and purified NIPA derivatives. The details of the data discussed in this section are presented on Figure 5.

The terminal cationic groups were formed in the polymer due to the presence of functionals added to the particle when dihydrochloride 2,2′-azobis (2-methyl propionamidine) AMP was used as the initiator. The cationic groups appeared as a result of proton attraction by the nitrogen of amino groups. The terminal anionic groups were revealed in the structure of the macromolecule in the case of the use of potassium persulfate KPS as the polymerization initiator [33]. The anionic groups occurred in the role of the donors of the protons. The comonomer N-(2-hydroxyethyl)acrylamide HEAA resembled cationic or anionic function, depending on the type of terminal groups in the macromolecule. The comonomer poly(ethylene glycol) dimethacrylate PEG-DMA had a faint influence on the level of protons in the aqueous environment. The differences in pH between N1 and N4 polymers came mainly from the equilibrium between the initiator and the main constituting polymer. The pH was definitively alkalic in N1, due to the presence of the terminal resembled by AMP, and definitively acidic in N4, due to the anionic terminals from KPS [34]. The PEG-DMA had a moderate impact on the final pH. The addition of HEAA to the reactant mixture resulted in the appearance of respective functionals within the structure of the particles. The acidity and the alkalinity of the systems of N3 or N6 were moderated. In the macromolecules synthesized with the anionic initiator, the groups from HEAA acted as proton acceptors, thus increasing the pH (N3). In the macromolecules synthesized with the cationic initiator and the addition of HEAA (N6), the pH decreased, presumably due to the donation of protons from the HEAA group to the functional groups from AMP [35]. The tendency was maintained in the case of systems N2 and N5 with a pH closer to the neutral (Figure 5).

#### 3.2.2. Electrokinetic Potential Below Volume Phase Transition Temperature

The variability in EP in the context of temperature was presented on Figure 5. Below VPTT, rather low EP values were observed, which should have led to the reduced stability of the macromolecule dispersions of the particles due to their low surface charge. Nevertheless, at these lower temperatures, the particles still formed a hydrated, macroscopically homogenous, colloidal system. The decrease or increase in pH coincided with the changes in electrokinetic potential, which gave us the excellent possibility to observe the phenomena of pH and particle charge in parallel. The decrease in the positive charge confirmed the decrease in the number of protons accepted from water. The less positive the particles were, the less protons were accepted from the aqueous environment, and the lower the pH was, as in the case of the following macromolecules: N4 > N6 > N5. The decrease in the module of negative charge was confirmative for the increasing pH: the negative values were less negative, due to the lower numbers of dissociating protons in the pattern: N1 > N3 > N2. For N6 and N3, the tendency of electrokinetic potential may confirm the assumption that the group from HEAA may be a donor or acceptor of protons.

#### 3.2.3. Electrokinetic Potential Above Volume Phase Transition Temperature

Conversely, at temperatures above the VPTT, local dehydration could occur, resulting in the predominant stabilizing effect of relatively high surface charge of the macromolecules, which dominated the stability of the colloidal system. Consequently, EP measurements obtained at 42 °C were of greater practical significance. The variations in EP values among particles of a similar composition, differing solely by the type of initiator, are minimal and exhibit a high degree of similarity at an increased temperature of 42 °C.

The temperature may be recognized as a factor, which definitively increases the electrokinetic potential. However, the increase depended both on the initiator, as well as on the comonomers. The most expressed electrokinetic potential change was recorded in samples synthesized with HEAA. The high change in the diameter could be ascribed to the high forces acting between the HEAA and the initiators. As it was presented for the pH, there is high possibility to exchange the protons between the functionals descending from the initiator and the HEAA. When heated, the activity of functional groups may increase, thus leading to the favored interchange of protons between donors and acceptors. The resulting complexes may have a character of insoluble salts, so the water should be repelled from the microgel, and the hydrophobic interactions may take place. Actually, the microgels in the constricted state have properties of insoluble particles and often its dispersions are not transparent.

### 3.3. Variability of the Hydrodynamic Diameter and Volume Phase Transition Temperature

#### 3.3.1. Hydrodynamic Diameter Below Volume Phase Transition Temperature

The measurements of the hydrodynamic diameter are of varied importance, due to the fact that there is a high possibility to obtain data influenced by the aggregation of the particles, or by the presence of specific functionals, which attract or repel water molecules. Although the obtained data are reasonable, the smallest structures were obtained in the case of N1 and N4, which may be a result of rigid, short cross-links, featured by relatively short PEG-DMA. This idea is supported by the small influence of increased temperature on the resulting hydrodynamic diameters of N1 and N4. The particles obtained with the mixture of PEG-DMA and HEAA (N3 and N6) increased its diameters, both when synthesized with KPS or with AMP as initiator. However, the increase was followed in the seria only by KPS, and the size pattern was N1 < N3 < N2. For the syntheses performed with AMP, the highest size was noted by N5, which could be ascribed to some repulsive forces between groups descending from HEAA and PEG-DMA, accompanied by AMP.

#### 3.3.2. Hydrodynamic Diameter Above Volume Phase Transition Temperature

**Figure 6:**
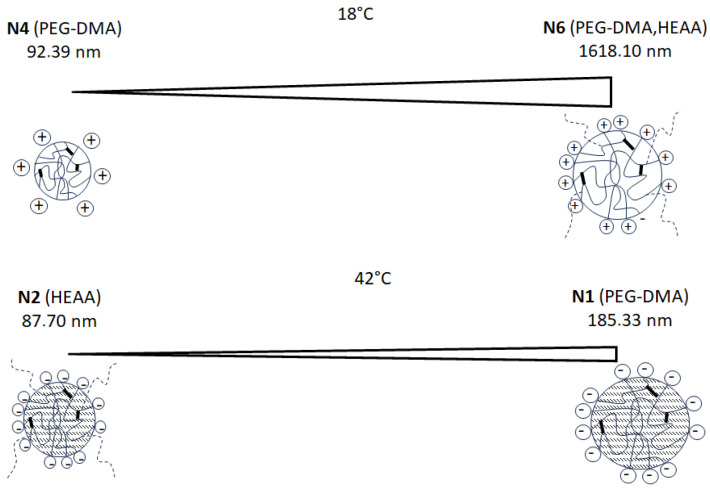
Schematic diagram showing composition and smallest and largest molecule sizes at 18 and 42 °C.

At higher temperatures, the absolute EP values increased, which indicated a high particle stability. The group of particles synthesized using HEAA had pH values in the range of 6, presumably because the hydrophilic HEAA comonomer has acidic properties. After exceeding the LCST temperature, the chains can adhere to the surface of the spheres, resulting in a large decrease in D_H_. EP values suggested the stability of the particles. When the hydrophilic monomer HEAA was used, very significant decreases in D_H_ were observed after exceeding the VPTT. The addition of HEAA resulted in the formation of branches outside the molecule. This was especially evident in the case of particles N5 and N6, where we observed a very large decrease in the value of D_H_. The addition of HEAA resulted in a significant increase in D_H_, and in the presence of the AMP initiator, the nitrites were positively charged and strongly repelled each other, resulting in a large particle size below VPTT. A diagram showing the composition and the smallest and largest molecular sizes at 18 and 42 °C is shown in Figure 6.

The D_H_ shows a general tendency to increase with temperature, suggesting possible particle swelling, aggregation, or changes in solvent–particle interaction dynamics. The consistently high PDI suggests that the particle population is not uniform, with multiple size populations potentially present. Temperature has a significant effect on particle size, probably due to thermal effects on the particle system. The high PDI suggests that processes, such as particle aggregation or disaggregation, may occur over the temperature range, resulting in a diverse size distribution. In order to optimize the system properties, efforts could be focused on reducing the PDI by adjusting the formulation or environmental conditions.

The value of VPTT was noticeably higher for polymers synthesized with the HEAA comonomer, with values ranging from 35 to 39 °C. In this manuscript, two methods are used to determine the VPTT values of the obtained particles. In the hydrodynamic diameter measurements performed in the previous assays [29,30,31], the applied wavelength of 678 nm was in the red region of the visible spectral range. In the spectrophotometer measurements, the applied wavelength of 480 nm was in the opposite region, the blue region of the visible spectral range. The difference between these two electromagnetic wavelengths did not influence the recorded VPTT value. This may be ascribed to the proximity of both wavelengths compared to the size and motion of the assessed particles. The obtained VPTT results were consistent when measured by both methods.

## 4. Materials and Methods

### 4.1. Materials

N-isopropyl acrylamide (NIPA, 97%, Sigma Aldrich, Steinheim, Germany), potassium persulfate (KPS, 98%, Sigma Aldrich, Steinheim, Germany), dihydrochloride 2,2′-azobis (2-methyl propionamidine) (AMP, granules, 97%, Sigma Aldrich, Steinheim, Germany), poly(ethylene glycol) dimethacrylate (PEG-DMA, Sigma Aldrich, Steinheim, Germany), and N-(2-hydroxyethyl)acrylamide (HEAA, Sigma Aldrich, Steinheim, Germany) were obtained from commercial sources and used without further purification. The dialysis bag with a molecular weight cut-off (MWCO) of 12,000–14,000 Da was supplied by Visking Medicell International Ltd. (London, UK). Deionized water conforming to the European Pharmacopoeia standards for purified water was obtained from an ionic column.

### 4.2. Methods

#### 4.2.1. Particle Synthesis

The assessed polymers were synthesized by SFPP, as it was presented in former works [29,30,31]. Shortly, the synthesis was carried out in a reactor consisting of a three-necked flask placed in a water bath, at a temperature of 70 °C, maintained and monitored by a temperature sensor immersed in the reacting mixture. The reactor was equipped with an Allihn reflux condenser and maintained under a nitrogen atmosphere. An appropriate volume of 70 °C deionized water was added to the reactor. The required initiators, KPS or AMP, were dissolved in 100 mL of deionized water and added to the reactor flask. Once the temperature of the mixture had stabilized, the remaining relevant reagents were dissolved in deionized water and added to the vessel containing the reactive solution. The composition of the synthesized particles is shown in Table 5.

After synthesis, a purification process was performed to remove unreacted monomers, crosslinkers, and initiators by equilibrium dialysis. The purification involved conductivity measurements of the acceptor liquid, deionized water, every 24 h until a constant conductivity value was reached. A dialysis membrane was used for this purpose. Conductivity measurements were performed using an ELMETRON CPC-511 (Gliwice, Poland) and an ELMETRON EC-70t conductive electrode (Zabrze, Poland). After completion of the cleaning process, the samples were freeze-dried in a Steris Lyophilizer Lyovac GT2 for further measurements. The molar ratio of monomer to initiator in compositions of N1–N6 microparticles is presented in Table 6.

#### 4.2.2. Hydrodynamic Diameter Measurements

The hydrodynamic diameters (D_H_) of the water dispersions of the purified particles were measured by dynamic light scattering (DLS) using a Malvern Zetasizer Nano ZS ZEN 3600 (Malvern Instruments Ltd., Malvern, UK) at a wavelength of 678 nm and an angle of incidence of 90°, using a polystyrene cuvette. Particle samples were diluted tenfold with deionized water. The polymer samples were evaluated using the backscattering measurement system at 173° and correlated with the parameters of the Mark–Houwink equation. Five measurements were taken for each sample, and the results were analyzed using Zeta Sizer Nano software version 5.03 (Malvern Instruments, Malvern, UK).

#### 4.2.3. pH and Zeta Potential and Measurements

The pH was measured using the potentiometric method with a CX-741 multifunction computer meter (ELMETRON, Zabrze, Poland) and an ERM-13-6 type electrode (ELMETRON, Zabrze, Poland) in the 250 mL conical flasks. Zeta potential (EP) measurements were performed in a Malvern Zetasizer Nano ZS ZEN 3600. Particle samples were diluted tenfold with deionized water. Five measurements were taken for each sample, and the results were analyzed using Zeta Sizer Nano software version 5.03 (Malvern Instruments, Malvern, UK). A DTS-1070 capillary cell dedicated for the EP measurement was used. Both measurements were performed in a temperature range of 18–42 °C in a nonbuffered aqueous solution.

#### 4.2.4. Volume Phase Transition Temperature (VPTT)

In this study, the VPTT was confirmed by spectrophotometric method. A UV-Vis 8453 spectrophotometer (Agilent, Santa Clara, CA, USA) equipped with a Brookfield TC-101 thermostat. Samples containing 3 mL of purified polymers solutions were prepared. Some samples were diluted tenfold with deionized water due to their intense opalescence. The absorbance of polymeric dispersions in thermally stabilized cuvettes was recorded in the wavelength range 190–1100 nm and temperature range 25–45 °C with 1 °C increments, and the values obtained at 480 nm were used.

## 5. Conclusions

The ionic equilibrium, in the evaluated polymeric systems of NIPA derivatives obtained from radical polymerization, is controlled by the dissociation of functional groups, which descend both from comonomers and the initiators. The process influences the pH of NIPA derivatives. The type of applied initiator, anionic or cationic, has an important role in the determination of the final charge of the pNIPA particles synthesized via SFEP. The pH and the electrokinetic potential may be assessed in parallel and used for extracting interesting information on the polymeric systems composed of micro- and nanoparticles, on the basis of NIPA. The PEG-DMA favors the synthesis of small microparticles of polyNIPA; whereas, the addition of HEAA results in the increase in the hydrodynamic diameter. Some repulsive and attractive forces between functional groups of the polymer may be observed and influence the recorded hydrodynamic diameter of synthesized NIPA derivatives.

## Figures and Tables

**Table 1:** Hydrodynamic diameter (D_H_) and polydispersity index (PDI) values of the evaluated particles.

Type of Initiator	Type of Polymer	D_H_ [nm]	SD	PDI	SD	D_H_ [nm]	SD	PDI	SD	Reference
		at 18 °C				at 42 °C				
Anionic	N 1 ^c a^	101.50	7.50	1.00	0.00	185.33	0.40	1.00	0.00	[29] ^a^ [30] ^b^ [31] ^c^
	N 2 ^a^	868.10	21.90	0.71	0.30	87.70	0.36	0.38	0.00	
	N 3 ^a^	483.20	16.43	0.63	0.09	115.90	4.84	0.42	0.02	
Cationic	N 4 ^b^	92.39	12.78	0.64	0.02	163.60	0.16	0.22	0.06	
	N 5 ^a^	1064.00	15.98	0.45	0.19	178.90	3.66	0.35	0.02	
	N 6 ^a^	1618.10	17.05	0.99	0.02	141.80	0.61	0.22	0.03	

SD-standard deviation, ^a,b,c^-these are references to specific publications where some important aspects of these particles have already been published.

**Table 2:** The pH values of the substrates used in particle synthesis in concentrations reflecting their usage in the reactant mixture.

Substrate	NIPA	KPS	AMP	PEG-DMA	HEAA
pH	6.23	4.23	5.86	5.95	6.23
SD	0.32	0.05	0.59	0.09	0.66

NIPA N-isopropyl acrylamide, KPS potassium persulfate, AMP dihydrochloride 2,2′-azobis (2-methyl propionamidine), PEG-DMA poly(ethylene glycol) dimethacrylate, HEAA N-(2 hydroxyethyl) acrylamide, SD-standard deviation.

**Table 3:** Zeta potential (EP) and pH values of the evaluated particles, BPP—before purification process, APP—after purification process.

Type of Initiator	Type of Polymer	EP [mV]				pH			
		at 18 °C	SD	at 42 °C	SD	pH_BPP_	SD	pH_APP_	SD
Anionic	N 1	−14.87	0.93	−28.97	0.50	2.43	0.02	3.42	0.06
	N 2	−4.97	0.94	−26.10	0.62	2.85	0.01	5.48	0.02
	N 3	−13.27	1.05	−21.1	3.17	2.86	0.02	4.62	0.04
Cationic	N 4	9.07	1.27	23.00	1.25	4.74	0.09	9.05	0.05
	N 5	−3.06	0.48	30.4	2.00	6.21	0.02	6.15	0.04
	N 6	1.68	0.19	26.23	2.64	6.12	0.02	6.25	0.06

SD-standard deviation.

**Table 4:** Volume phase transition temperature (VPTT) of the evaluated particles.

Type of Initiator	Type of Polymer	VPTT [±0.5 °C]
Anionic	N 1	32–34
	N 2	35
	N 3	38
Cationic	N 4	32–34
	N 5	36
	N 6	37

**Table 5:** Qualitive and quantitative composition of the evaluated particles.

Substrates % (*w*/*w*)	Monomer	Initiator		Crosslinker	Comonomer	Solvent	Bibliography *
		Anionic	Cationic	Long Chain	Hydrophilic		
Type of Polymer	NIPA	KPS	AMP	PEG-DMA	HEAA	H_2_O	
N 1 ^a^	0.5	0.05		0.05		99.40	[29] ^a^ [30] ^b^ [31] ^c^
N 2 ^c^	0.5	0.05			0.05	99.40	
N 3 ^c^	0.5	0.05		0.05	0.05	99.35	
N 4 ^b^	0.5		0.05	0.05		99.40	
N 5 ^c^	0.5		0.05		0.05	99.40	
N 6 ^c^	0.5		0.05	0.05	0.05	99.35	

NIPA N-isopropyl acrylamide, KPS potassium persulfate, AMP dihydrochloride 2,2′-azobis (2-methyl propionamidine), PEG-DMA poly(ethylene glycol) dimethacrylate, HEAA N-(2 hydroxyethyl) acrylamide, H_2_O deionized water. * Polymer compositions from previous studies that have already been published by our scientific group. In this paper, selected physicochemical studies of synthesized particles are expanded in the field of electrokinetic potential. ^a,b,c^-these are references to specific publications where some important aspects of these particles have already been published.

**Table 6:** The molar ratio of monomer to initiator in compositions of N1–N6 microparticles.

Type of Polymer	Monomer (mol)	Anionic Initiator (mol)	Cationic Initiator (mol)	NIPA to KPS/AMP **Radical Molar Ratio**
	NIPA	KPS	AMP	
N1–N6	4.4 × 10^−2^	1.85 × 10^−3^	1.84 × 10^−3^	1:0.1

## Data Availability

Data is contained within the article.
